# The Flame Retardancy of Polyethylene Composites: From Fundamental Concepts to Nanocomposites

**DOI:** 10.3390/molecules25215157

**Published:** 2020-11-05

**Authors:** Erfan Rezvani Ghomi, Fatemeh Khosravi, Zahra Mossayebi, Ali Saedi Ardahaei, Fatemeh Morshedi Dehaghi, Masoud Khorasani, Rasoul Esmaeely Neisiany, Oisik Das, Atiye Marani, Rhoda Afriyie Mensah, Lin Jiang, Qiang Xu, Michael Försth, Filippo Berto, Seeram Ramakrishna

**Affiliations:** 1 Center for Nanofibers and Nanotechnology, Department of Mechanical Engineering, Faculty of Engineering, National University of Singapore, Singapore 117576, Singapore; fatemeh_khosravi22@yahoo.com (F.K.); zhr.mossayebi@gmail.com (Z.M.); 2 Department of Polymer Engineering, Faculty of Engineering, Golestan University, P.O. Box 491888369, Gorgan 1575949138, Iran; alisaedi2012@yahoo.com; 3 Department of Polymer Engineering and Color Technology, Amirkabir University of Technology, P.O. Box 15875-4413, Tehran 1591634311, Iran; morshedi.iut91@gmail.com; 4 Department of Chemistry, Materials and Chemical Engineering “G. Natta”, Politecnico di Milano, Piazza Leonardo da Vinci 32, 20133 Milan, Italy; masoud.khorasani@mail.polimi.it; 5 Department of Materials and Polymer Engineering, Faculty of Engineering, Hakim Sabzevari University, Sabzevar 9617976487, Iran; r.esmaeely@hsu.ac.ir; 6 Structural and Fire Engineering Division, Department of Civil, Environmental and Natural Resources Engineering, Luleå University of Technology, 97187 Luleå, Sweden; michael.forsth@ltu.se; 7 Baspar Sadaf Nab Sepahan, between 23 and 24 Streets, Mahmoodabad Industrial Town, Isfahan 8161199774, Iran; atiye.marani@gmail.com; 8 School of Mechanical Engineering, Nanjing University of Science and Technology, Nanjing 210094, China; ramensah@ymail.com (R.A.M.); ljiang@njust.edu.cn (L.J.); xuqiang@njust.edu.cn (Q.X.); 9 Department of Mechanical and Industrial Engineering, Norwegian University of Science and Technology NTNU, S.P. Andersens Veg 3, 7031 Trondheim, Norway; filippo.berto@ntnu.no

**Keywords:** flame retardancy, polyethylene, intumescent, fire, flammability

## Abstract

Polyethylene (PE) is one the most used plastics worldwide for a wide range of applications due to its good mechanical and chemical resistance, low density, cost efficiency, ease of processability, non-reactivity, low toxicity, good electric insulation, and good functionality. However, its high flammability and rapid flame spread pose dangers for certain applications. Therefore, different flame-retardant (FR) additives are incorporated into PE to increase its flame retardancy. In this review article, research papers from the past 10 years on the flame retardancy of PE systems are comprehensively reviewed and classified based on the additive sources. The FR additives are classified in well-known FR families, including phosphorous, melamine, nitrogen, inorganic hydroxides, boron, and silicon. The mechanism of fire retardance in each family is pinpointed. In addition to the efficiency of each FR in increasing the flame retardancy, its impact on the mechanical properties of the PE system is also discussed. Most of the FRs can decrease the heat release rate (HRR) of the PE products and simultaneously maintains the mechanical properties in appropriate ratios. Based on the literature, inorganic hydroxide seems to be used more in PE systems compared to other families. Finally, the role of nanotechnology for more efficient FR-PE systems is discussed and recommendations are given on implementing strategies that could help incorporate flame retardancy in the circular economy model.

## 1. Introduction

In recent times, polymers have been used in a wide range of industrial applications, such as packaging, electronics, and construction [1,2]. To better the performance of the aforementioned applications, polymers need to be more environmentally friendly, safe, and durable [3]. The utilization of polymers is encouraged due to good functionality, acceptable durability, and cost-efficiency; however, high flammability, and poor thermal properties are noticeable limitations for their applicability [4]. Most polymers are flammable and, by exposure to enough energy, they will leave a considerable amount of smoke and heat [5]. It is reported that annually about 5000 people in Europe and 4000 people in the United States are killed by fires. Furthermore, the economic loss of these fire accidents is estimated to be about 0.3% of the GDP [6]. Following the analysis conducted by Geneva-based Aircraft Crashes Record Office (ACRO), it is possible to reduce the possibility of fire catastrophes and their subsequent fatalities by reducing the flammability of polymeric materials [6]. Therefore, related to the use of polymers, there are environmental, economic, and health issues which are considered as the driving forces to motivate the conduction of research and different studies on the flammability of polymers [7].

Polyolefins are a group of the most popular polymers in various applications [8]. One of the main polymers of this group is PE, with more 100 million tons production per year, that is, 34% of total plastic market, and is extensively used due to good mechanical and chemical resistance, low density, cost efficiency, ease of processability, non-reactivity, low toxicity, good electric insulation, and good functionality [9]. However, like other polymers, the concern about PE is its flammability in applications requiring good flame retardancy. Although the emissions from PE have low toxicity, it has a low limiting oxygen index (LOI) and drips during burning, which causes rapid flame spread [10].

Typically, physical or chemical modifications, as well as applying FRs, are recommended to reduce the flammability of PE products [11]. Flame retardancy of polymers increases by incorporation of FRs and is estimated to triplicate their survival time in case of fire [12]. FRs are necessary as they enhance the flammability of polymeric materials by delaying the fire and it’s spread [13]. The common commercial FRs used for PE systems are based on phosphorus, borate, inorganic hydroxides, silicon, and nitrogen and are introduced to polymer matrixes during processing [14]. Each of the FR approaches uses a different mechanism and their application is attributed the polymer matrix and the intended usage. For example, regarding the inorganic hydroxides, they will reduce the burning rate by releasing water to decrease the temperature since decomposition occurs at higher temperatures. Although gas-phase reactions are important in controlling flames in PE systems, quenching FRs based on halogens are not recommended due to environmental concerns [15]. Moreover, intumescent flame retardants (IFRs) are of great importance and efficiency in restricting the fire by forming a swollen char and are applicable for polymeric materials [16]. Considering the high production and applicability of PE, the issue of its flammability is of great importance and hence, there is an urgent need to develop strategies that minimizes the flammability of PE.

A web search revealed that several review papers have been published on flame retardancy of polymers; however, there is a dearth of comprehensive research on FRs for PE composites [16,17]. Herein, this study reviews the specific approaches in enhancing flame retardancy of PE and some research papers published in the past 10 years, whiletaking into account the newly emerged FR nanocomposites.

## 2. PE Grades and Properties

PEs are one of the most potential materials of value-adding in case of accurate formulation modifications [18]. It is of great importance to know the grades of PE as their flame performance is correlated with their chemical structure (i.e., branching type) and FR formulations [18]. The structure and properties of PE main grades, including density, crystallinity, LOI, thermal conductivity, melting temperature, and molecular weight are summarized in Table 1.

## 3. FR Approaches and Materials

In this section, the specific approaches and materials used for enhancing the flame retardancy of the PE systems are discussed and their recent works are investigated.

### 3.1. Phosphorous and Melamine

Phosphorus-based FRs are the most considerable halogen-free type of FRs used for improving flame retardancy of PE. Common phosphorus-based FRs include red phosphorus (RP), phosphine oxides, phosphines, phosphonates, phosphates, ammonium phosphate, and phosphites [19,20]. Regarding the PE structure, phosphorus compounds are more advantageous in comparison with the halogen-based FRs as they work in two separate phases, gas and condense [21]. The physical and chemical reactions mostly affect flame inhibition and heat reduction because of the controlling melt flow, surface protection by acids, char layer promotion, and char layer protection against oxidation. Phosphorus FRs volatilize into the gas phase and strongly scavenge the hydrogen and hydroxyl radicals [22]. The Equations (1)–(5) demonstrates the radical scavenging of Ḣ and ȮH by the active phosphorus radicals such as PȮ and HṖO_2_, which are present in the flame (M is a third body species) [21].

(1) $$HP{\dot{O}}_{2}+\dot{H}\to \mathrm{PO}+H_{2}O$$

(2) $$HP{\dot{O}}_{2}+\dot{H}\to {\mathrm{PO}}_{2}+H_{2}$$

(3) $$HP{\dot{O}}_{2}+O\dot{H}\to {\mathrm{PO}}_{2}+H_{2}O$$

(4) $$P\dot{O}+\dot{H}+M\to \mathrm{HPO}+M$$

(5) $$P\dot{O}+O\dot{H}+M\to {\mathrm{HPO}}_{2}+M$$

The produced HPO can effectively quench the flame and lower the reactivity of the material compared to Ḣ and OḢ. Some phosphorus-containing compounds can decompose to phosphoric acid and polyphosphoric acid in the condense phase. A molten viscous layer formed by acids protect the surface of the polymers and restrict oxygen penetration [22,23]. Ammonium polyphosphate (APP) has been broadly used in IFR systems as an acidic source and blowing agent. Khanal et al. [24] prepared a novel IFR system containing APP and tris (2-hydroxyethylene) isocyanurate (THEIC) to enhance the fire and flammability performance of HDPE. Flammability of the prepared HDPE/IFR (AAP/THEIC) composite was evaluated by LOI analysis and cone calorimeter tests (CCT) indicating different parameters, but just one of them is investigated, which is peak heat release (PHR) rate here. The LOI analysis indicated that the LOI value of the HDPE/IFR composite with the weight ratio of 3:1 was higher than the LOI value of the pure HDPE and HDPE/APP. It is evident that adding THEIC as a char agent increased the flame retardancy of HDPE/IFR composite while the most striking feature was the optimum weight ratio of APP (3) to THEIC (1). The LOI value of the composite decreased by increasing the amount of THEIC. The CCT analysis illustrated that the combustion time of HDPE/IFR composite is slower than the pure HDPE and the PHR rate decreased and occurred at a longer time. It is clear that the charring formation attributing to THEIC component caused a significant reduction of PHR rate value.

**Figure 1:**
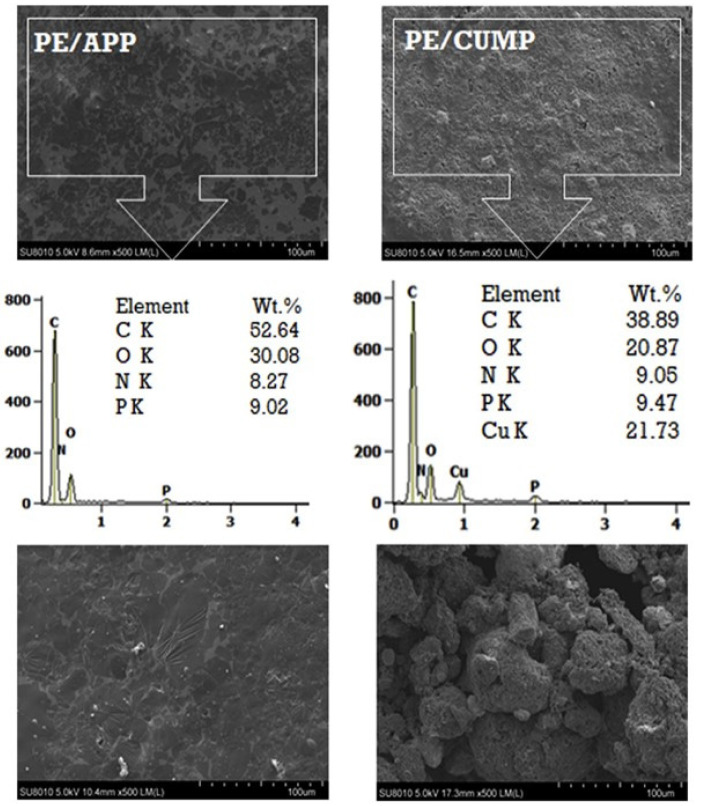
SEM images of cone calorimetry residues of Polyethylene/Ammonium polyphosphate (PE/APP) (outer and inner) and Polyethylene/Copper phosphate and melamine phosphate (PE/CUMP) (outer and inner), reprinted with permission from Ref. [4].

Melamine (MLM) is another unique material containing 67 wt.% nitrogen and excellent thermal resistance, which could be combined with phosphorus compounds used in FR applications. Among the MLM-containing compounds, MLM phosphate is specific due to the presence of phosphorus. Other commercially available MLM-based FRs are melamine cyanurate (MC), melamine pyrophosphate (MP), and melamine polyphosphate (MPP). Moreover, MLM can form salts with high thermal stability and strong acids. Salasinksa et al. [4] evaluated the effect of incorporating copper phosphate and melamine phosphate (CUMP) into HDPE as a FR compound and compared it with HDPE/APP. The fire property of HDPE containing CUMP was carried out by CCT. Figure 1 indicates the scanning electron microscope (SEM) images of PE/APP and PE/CUMP formed char layer, as well as char chemical composition.

The SEM images of the residues CCT conducted on HDPE/APP and HDPE/CUMP samples confirmed the char formation of both. Regarding the HDPE/APP, the formed char layer was thin without swollen structure while HDPE/CUMP was found to be porous inside char. The chemical composition analysis demonstrated that the char layer composed of C, O, N, and P components. CUMP were likely to be decomposed to CuO and P_2_O_7_, and then take part in the crosslink process, which finally causes a dense char layer formation and mechanical behavior improvement. It was evident that the HDPE/CUMP resulted in more char formation in comparison with pure HDPE. Compared to pure HDPE, HDPE/CUMP showed a significant reduction in PHR rate. Furthermore, the CUMP worked in both condense and gas-phase via char layer formation and emission of more non-flammable gases, respectively. Other works on the flame-retardancy of PE using phosphorous and MLM compounds have been summarized in Table 2.

### 3.2. Nitrogen

Nitrogen-based FRs have also been used in PE systems to improve its flame resistance. Essential environmentally friendly groups of FRs are nitrogen-comprising ones because of their low toxicity, efficiency, recyclability, and their low evolution of smoke during combustion [47,48]. Commonly known nitrogen-based FRs are ammonia, MLM, and their derivatives, whilst other types based on urea and guanidine are also identified. Generally, MLM and derivatives are the basic components of an intumescent system [49,50]. Nitrogen-containing compounds showed a well-effective synergism with the fire retardants containing phosphorus [51]. As a case in point, MP and APP materials, taking advantage of N-P synergism, are the most used P and N-containing fire retardants. Besides, MLM/ammonium salts with organic or inorganic acids such as boric acid (BA), cyanuric acid, phosphoric acid, or pyro/poly-phosphoric acid are typically used to provide higher thermal stability along with lower volatility [52]. Polymeric nitrogen compounds based on cyanuric acid have been recently developed and MC, as a two-dimensional, highly thermally stable structure, showed strong synergism with phosphorus compounds [53,54].

**Figure 2:**
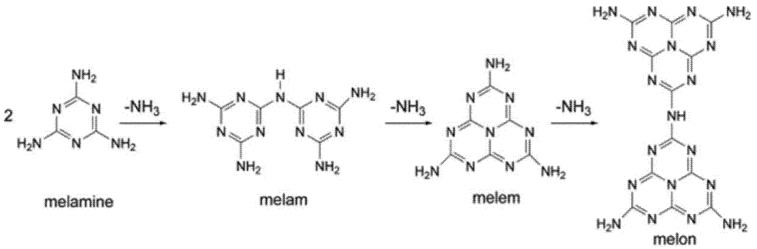
Chemical structure of the condensates produced through endothermic thermal decomposition of the melamine, reprinted with permission from Ref. [55].

The flame retardancy action mode of MLM and its salts is somewhat different [55]. Upon heating of the pure MLM, MLM starts volatilization by heat absorption caused by cooling down the surface of the polymeric matrix. Under high temperature, MLM undergoes further endothermic decomposition to produce cyanamid [56]. In the meanwhile, thermally stable condensates, i.e., melam, melem, and melon, as well as ammonia gas, may form. The chemical structure of the produced condensates is illustrated in Figure 2. These residue condensates contribute to the creation of an insulative layer in the condensed-phase. In addition, the ammonia evolution dilutes the burning atmosphere with inert/non-combustible gases, causing a gas phase flame retardancy contribution. In MLM salts, if MLM reforms upon dissociation of the salts exposed to the heat, a same decomposition mechanism is expected. Because of more progressive condensation in MLM salts rather than the pure MLM, their condensed-phase contribution is dominant [57]. Likewise, N-containing synergistic systems either form char through condensed-phase or promote gas phase reaction to scavenge free radicals [52,58]. Moreover, the ammonium salts decrease the spread rate of the flame but evolve potentially toxic gases [58].

In an interesting study reported by Luyt et al. [18], six FR compositions obtained by mixing commercial N- and P-containing FRs were embedded into different grades of LDPE and LLDPE to evaluate their effects on thermal stability and fire-resistance. LDPE grade produced in an autoclave reactor showed the best flame retardancy performance (UL 94 V0, char residue: 10 ± 1% at 800 °C) when 35 wt.% of the triazine (TRZ) derivative and APP formulated FR was incorporated. This was due to the phosphorus-nitrogen synergism forming the highly thermally stable phosphorous oxynitride residue. In contrast, LDPE from the tubular reactor and LLDPE, contained TRZ derivative and APP formulated FR, represented poorer fire resistance since their strongly entangled molecules hindered well dispersion of FRs and lessened their activity due to the premature thermal decomposition. Results of the nitrogen-based FR revealed that nitrogen-based compounds alone could not achieve a V0 rating in any grades of PE. Table 2 indicates the works conducted by using nitrogen-based FRs in PE systems during the past 10 years.

The nitrogen and phosphorus-based flame retardants used as treatments in PE, LDPE, LLDPE, and HDPE are listed in Table 2 with emphasis on the effect each additive have on the polymer matrix. Meanwhile, some additives greatly improve the flame retardancy of the polymeric composites others, such as the Phenyl phosphinic arid di-4-[1-(4-pheny phodphonic acid monophenyl ester-yl)-methyl-ethyl] phenyester dimelaminium (PDEPDM), caused a reduction in the mechanical properties of PE. Hence, it is more appropriate to apply FRs that enhance fire properties without compromising their mechanical properties.

### 3.3. Inorganic Hydroxides

Inorganic hydroxides are widely applied to develop the fire retardancy of PE products because of their benefits, such as low toxicity, cost efficiency, minimal corrosion, and contribution to declining smoke emission during the combustion process. In addition, releasing water at the temperature above 200 °C is a distinct characteristic of inorganic compounds. The two main types of inorganic hydroxide are ATH and magnesium hydroxide (MH). There are typically two mechanisms of action in flame retardancy of ATH and MH compounds including flame dilution and the catalytic effect, which leads to charring enhancement [59]. The anhydrous alumina and magnesia are white powerful refractor powders, which can reflect the heat and assist to improve heat insulation with aggregating on the surface. Regarding the ATH, water releasing occurs at 220 °C, while MH releases water at 330 °C. The largest commercially use of ATH and MH FRs is in wire and cable insulation applications. There are several advantages associated with ATH, such as low cost, non-toxicity, and excellent flammability behavior. Generally, flame retardancy analysis requires at least 35% of metal hydroxide. Increasing the amount of metal hydroxide could lead to degrading physical properties as well as low-temperature flexibility. As a result, one of the most important techniques is to combine metal hydroxides with other FRs, such as phosphorus compounds, boron compounds, and nanoclays [60,61]. In some cases, surface modification is effective to increase flame retardancy of hydroxide compounds. Moreover, ATH is able to work in two different phases, gas and condense. The main mechanism of ATH in the condensed phase is heat absorption during the decomposition process. The ATH decomposition occurs at the range of 220–400 °C based on the reaction as shown in Equation (6) [62].

(6) $$2\mathrm{Al}{\left(\mathrm{OH}\right)}_{3}\to {\mathrm{Al}}_{2}O_{3}+3H_{2}O$$

The decomposition reaction of ATH is extremely endothermic and absorbs about 1kJ/gr heat. The heat absorption reaches its maximum at 300 °C. The most striking feature is the formation of water vapor from the hydroxyl groups bonded to aluminum. Furthermore, the combustion is hindered by releasing the water vapor into the fire, diluting the flammable gases concentration, and limiting the accessibility of oxygen to the surface of the composite [62]. On the contrary, to the halogenated-FRs, the produced gases from the decomposition reaction of ATH are non-toxic and non-corrosive. Generally, the required characteristics of inorganic hydroxides FRs in commercial products are: (a) low cost, (b) highly accessible with low surface area and small particle size, (c) low toxicity, (d) exhibiting endothermic decomposition reaction between 100–300 °C, (e) capable of being used at high loading, and (f) colorless [22].

Layered double hydroxides (LDH) are synthetic materials containing negatively charged layers of inorganic/organic anions, which alternately located in the interlayer of positively charged layered of metal hydroxide [63,64]. The general formula of LDH is presented in Equation (7) [64].

(7) [M1−x2+Mx3+(OH)2]x+Axnn−yH2O

LDH is considerably important to be used in commercial polymer industries, as they are environmentally friendly FRs. These types of FR compounds are attractive due to their endothermic decomposition against high temperature, nontoxicity, and high level of smoke suppression [64,65]. Until now, various studies have been conducted to evaluate the effect of incorporating metal hydroxides with polymers and nanocomposites.

**Figure 3:**
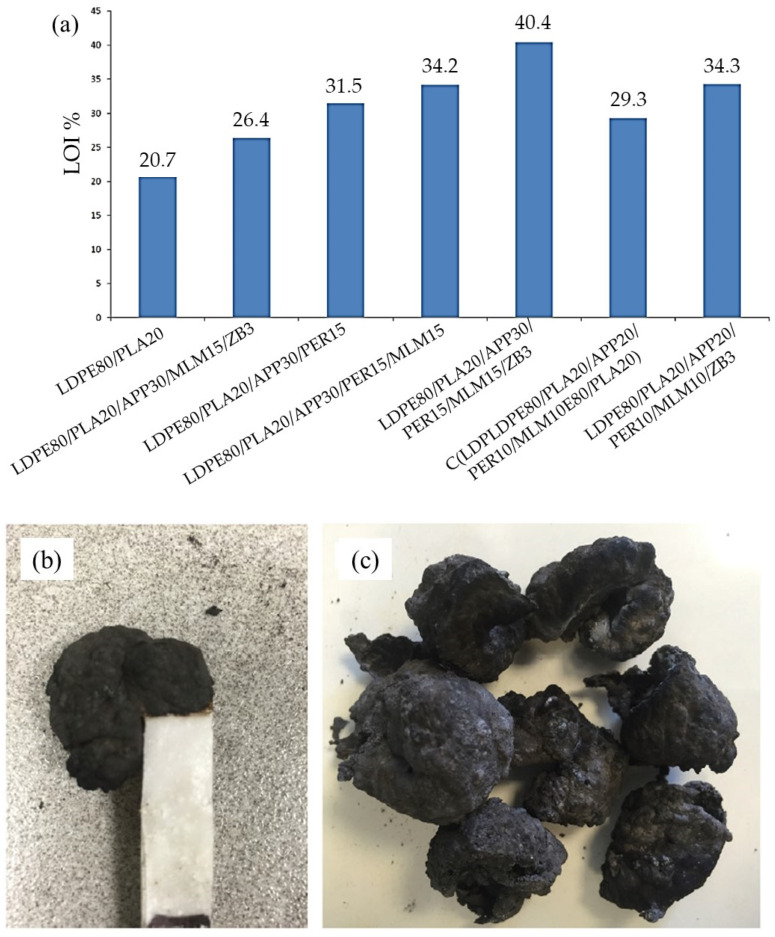
(**a**) Limiting oxygen index (LOI) values of all samples, images of (**b**) char of the LDPE/PLA/APP/MLM/PER after the LOI analysis, and (**c**) char residue of the LDPE/PLA/APP/MLM/PER, reprinted with permission from Ref. [66].

Arslan et al. [66] investigated the result of using metal hydroxide, ZB, MLM, APP, and PER as reinforcements to improve flammability of LDPE/Polylactic acid (PLA). The efficiency of flame retardancy of the composites was evaluated by LOI analysis. The results showed the higher LOI values for samples containing MLM, APP, and PER. The LOI values of LDPE/PLA/ZB, LDPE/PLA/AH, LDPE/PLA/MH, LDPE/PLA/MLM, and LDPE/PLA/APP were 21.3, 20.2, 21.4, 20.4, and 22.3, respectively. As can be observed, the compound of ZB, AH, MH, and MLM indicated a negative impact on flammability of the LDPE/PLA blend. The highest value of LOI analysis was attributed to LDPE/PLA/APP30/PER15/MLM15/ZB3 composite with 95.17% improvement in comparison to the LDPE/PLA blend. The best flame retardancy results were reported in Figure 3a. It is crystal clear that APP is the most effective FR, which significantly promoted the flame behavior of LDPE/PLA. In addition, APP/MLM/PER incorporation considerably increased the strength and integrity of the char layer due to the promotion of phosphor and oxygen. Figure 3 demonstrates the images of char layer after LOI analysis and char residue of LDPE/PLA/APP/MLM/PER composite.

Recent studies on the flame-retardancy of PE using inorganic hydroxides have been summarized in Table 3.

From Table 3, Huntite and hydromagnesite caused a reduction in the tensile strength and elongation of the LLDPE material. Some of the notable additives that must be taken into consideration with regards to the flame retardancy of PE and its grades are the Organopalygorskite (OPGS), Molybdenum sulfide (MoS_2_), and MH, which all resulted in a drastic reduction of peak heat release rate. Furthermore, the addition of MH, TiO_2_ and LDPE composites had an excellent fire resistivity and mechanical performance. Aside from Modified MH, all the additives of HDPE produced better results and can therefore be utilized when developing flame retardant strategies for PE.

### 3.4. Boron

In addition to the water-soluble boron compounds including sodium borate (borax), BA, and boron oxide, water-insoluble ones and more commonly ZBs, are widely used as boron-derived FRs, former for application in cellulosic materials and latter in thermoplastics [52]. Boron based FRs possess low cost, high thermal stability, non-toxicity, and ease of handling, which have resulted in their wide use in PE systems [90]. Employment of ZB, with the most commercial importance, not only improves flame retardancy but also helps with the smoke suppression and anti-arcing in condensed phase [52]. Although ZB FRs are often used in halogen-containing systems, it has also been utilized in halogen-free and FR polymers [91]. Based on the studies conducted by Li et al. [92] and Wu et al. [93], the introduction of ZB into PE systems can result in the increase in residual char and thermal stability improvement. An extensive review of various types of ZB and their application was conducted recently by David M. Schubert [94]. In the most reported literatures [95,96], boron compounds were used together with other synergistic retardants such as nitrogen, phosphorus, silicon, and other synergies that has been comprehensively investigated in the following section. Therefore, other boron-based FRs, including calcium borate, melamine borate, boron phosphate, ammonium pentaborate, borosiloxane, etc., have become potential candidates only in recent years [52].

Upon heating and polymer combustion, depending on the grades of ZBs, endothermic dehydration occurs, in which the ZB loses its chemically bonded water molecules. This water vaporization not only provides a heat sink, delaying the combustion, but also dilutes the concentration of the oxygen and gaseous flammable components in the flame zone, causing an enhancement in the residual char formation [97]. Furthermore, at elevated temperatures, a glassy protective layer may form on the polymer/char surface, acting as a barrier to the transfer of heat, oxygen, and decomposition products, resulting in char strength and further combustion retardant [98]. Hence, another function of the ZB would be inhibiting the oxidation of the char (afterglow suppression), as well as suppressing smoke formation. Accordingly, a change in the oxidative decomposition direction of the halogen-free polymers (e.g., PE) is demonstrated when ZB is used. However, it is hesitated due to the suppression effect of boron oxides on hydrocarbons’ decomposition [99], or graphite oxidation in the char [59], or just because of the insulative layer formation. Besides contributing to the condensed phase retardancy, in the presence of halogens, a gas phase flame retardancy is also attributed to the ZB due to the production of halogenated compounds, scavenging hot radicals, during the reaction between ZB and halogens. It is believed that BA and ZB are following the same flame retardancy mechanism just a few studies on flame retardancy effects of BA are released [100,101]. On the other hand, boron oxide can function only in the condensed phase by forming an insulative layer [52].

**Figure 4:**
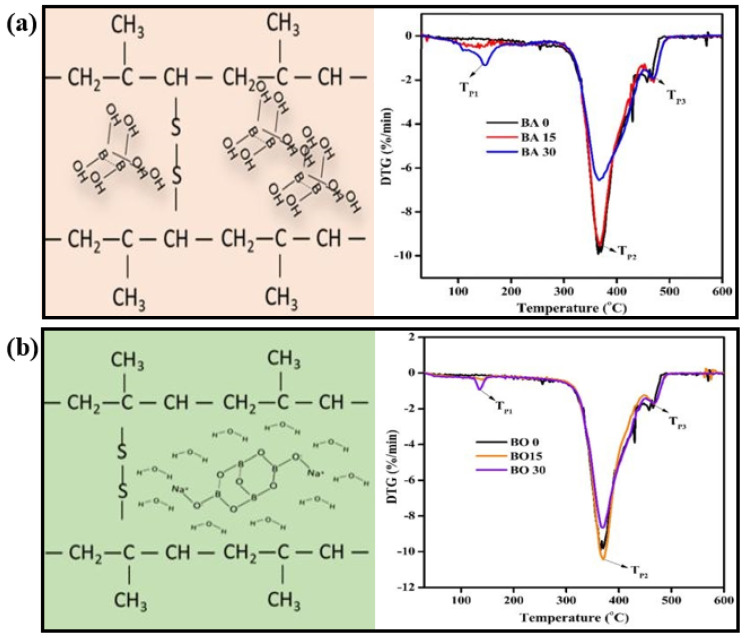
Effect of the boron compound additive on cross-linking and DTG curves of the NR/LDPE/HAF (100/10/30) with varying loadings (0–30) of (**a**) BA, and (**b**) borax (BO), reprinted with permission from Ref. [103].

Recently, Sultigova et al. synthesized a certain chemical composition of ZB (2ZnO•3B_2_O_3_•3,5H_2_O), using borax and zinc sulfate as the precursors in aqueous solution, aiming at producing composites based on HDPE [102]. The composites were obtained via extrusion of the mixture at several prescribed temperatures. It is found out that the polymeric composites burnt much more slowly, without the formation of polymer melt droplets. Furthermore, the percentage of LOI and coke residue (CR) of the composites was 20.6 and 8.6, respectively, which were much higher than those values for the initial polymer. The results revealed that the incorporation of ZB into HDPE, increased the fire resistance of the starting polymer without diminishing its mechanical properties. Another recent study is developed by Abdulrahman et al. to investigate the effect of BA and borax on the thermal and viscoelastic properties of natural rubber (NR)/LDPE/high abrasion furnace carbon black composites [103]. For both fillers, the residual char yield was increased in the related composites. The loading of the fillers showed a considerable impact on the flammability behavior of the composites altering it from slow-burning to self-extinguishing (LOI: 28.5%) for BA and to the upper range of slow-burning (LOI: 27.8%) for borax (Figure 4). Boron-containing FRs can show an advantageous synergistic interaction with MH, phosphorus, carbon, Si- and N- comprising compounds. For instance, Boron phosphate or metal borophosphate are produced in the intumescent systems containing phosphorus compounds (i.e., APP) and BA or ZB and boost the char formation and integrity. In the case of boron-nitrogen synergies, generation of boron nitride during fire may change the dominant fire retardancy mode of action. Furthermore, for the boron-nitrogen synergetic systems, it is believed that borosilicate glass or ceramic is formed because of borate/silica fusion at high temperatures. This phenomenon increases the fire resistance in the condensed phase [97]. Other works in the past 10 years on the flame-retardancy of PE using boron compounds have been summarized in Table 4.

### 3.5. Silicon

Silicon-containing FRs always have been in the frontline of co-additives in FR systems for PE products due to their high versatility, compatibility, low toxicity, and environmentally friendly characteristics [47,97]. This class of compounds can be mainly categorized into silicones, silica, organosilanes, silsesquioxanes, and silicates, functioning in FR systems as additives or other forms [52,90]. Silicon-comprising materials are inherently thermal stable and during a fire, they can produce an insulative layer upon decomposition. Through the formation of a highly thermally stable char, further substrate decomposition would be suppressed, and the rate of heat release would be lowered. Thus, the combustion of silicones is only associated with the emission of a negligible amount of toxic gases and smoke [124]. The reduction of HRR, PHR rate in CCT, and the rate of combustibles are most evident in silicone and siloxane. Besides fire retardancy through the condensed phase, the functionalization of silicone-based FRs with phosphorous or nitrogen groups makes them more efficient through contribution to the gas phase by trapping of dynamic radicals in the vapor phase [90,125].

**Figure 5:**
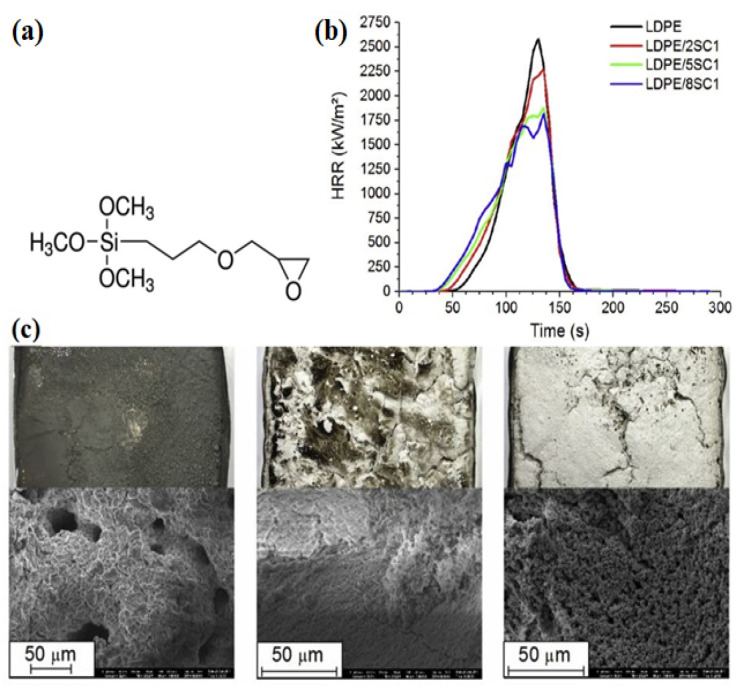
(**a**) Chemical structure GOPTMS; (**b**) Burning behavior of the LDPE-based systems in which various loadings of SC1 incorporated; and (**c**) Surface and microscopic structure of the fire residue in LDPE/SC1, LDPE/MH, and LDPE/SC1/MH composites (from left to right), adapted with permission from Ref. [81].

Silicon dioxide, known as silica, in forms of silica gel, fumed and fused silica, is the most common silicon-based FR tested in various polymeric matrixes. Employment of functionalized silica and nano-silicates, in which oligomers or polymers attached through silanol groups, is a promising approach to produce the most efficient silicon-based fire retardants. This group of organic silicon compounds attracted intensive attention in recent years and the amount of research introducing these novel additives is growing [81,110]. Scarfato et al. reported the incorporation of a novel SC1 into the LDPE matrix to investigate its thermal and burning behavior together with its synergism with MH [81]. The SC1 was synthesized through the functionalization of MMT with (3-glycidyloxypropyl) trimethoxysilane (GOPTMS) by a silylation procedure (Figure 5a). LDPE/SC1, LDPE/MH, and LDPE/SC1/MH composites with various loading of the fillers were prepared. For binary LDPE/8SC1 system, the LOI value increased only from 17.5 up to 18.6 vol%, demonstrating a minor change that may be still within the margin of uncertainty. This is probably because of inadequate protection against direct contact to flames, provided by the inorganic residue layer formed by nanoclays. On the other hand, the addition of SC1 to LDPE/MH composites lowered their LOI indicating an adverse effect on the flame retardancy of the system, which is likely due to the worsening of the protective fire residue quality. Furthermore, with increasing SC1 content in LDPE/SC1 composites, the time to ignition (TTI) was decreased (Figure 5b).

In addition to the aforementioned organic silicones, noticeable research works have been conducted recently on polydimethylsiloxane (PDMS), as one of the most important polyorganosiloxanes, to modify the fire-resistance properties of organic polymers, through direct mixing with the polymers, deposition of PDMS on the fillers or synthesis of copolymers [126]. Owning to limited thermodynamic miscibility of PE with PDMS, ethylene-methyl acrylate copolymer (EMA) is often used as the chemical compatibilizer in a LDPE-PDMS mixture [127]. A recent review on flame resistance of PDMS systems by Zielecka et al. is a comprehensive reference for more information [124]. Moreover, polyhedral oligomeric silsesquioxane (POSS) with their specific hybrid organic-inorganic structures, also attracted significant attention in recent years [128]. An overview of the fire retardancy properties of polymer/POSS nanocomposites is represented by Zhang et al. [129]. Monofunctional POSS can contribute to the polymerization processes to produce, for instance, PE-POSS, poly(methyl methacrylate)-POSS, or other nanocomposites [130]. Whilst the addition of 2.5 wt.% octamethyl POSS into the PE–calcium carbonate–silicone (CaSiEMAA) composite system showed poor performance in the CCT, it eliminated dripping completely which is likely because of promoting ceramization of the silicon at the surface [121]. In essence, the rich chemistry of silicon compounds, especially, their inherent thermal stability, makes them strong candidates for FR applications. Table 4 shows a summary of recent studies in the past 10 years on PE systems using boron-based FRs.

Table 4 shows that POSS can reduce the dripping effect of PE. Nano clays are effective in extending the ignition times and reducing fire growth capacity, PHR rate as well as increasing the mechanical properties of PEs. The presence of ZB enhances the crystallinity of PE whiles an increase in the ratio of BA/BX has an adverse effect on ignition time, HRR, smoke production rate. It is therefore of great importance to maintain the blends at desirable ratios.

## 4. The Role of Nanotechnology in Flame Retardancy of Polymer Nanocomposites

Nowadays, the most important role of nanomaterials in polymer nanocomposites is the improvement of mechanical properties, such as impact strength and stiffness [131,132]. First, the employment of nanocomposites as FRs is receiving great attention because of nanomaterials with high aspect ratio, and many studies are conducted in this field [133]. Although some of the research in this field indicated improvement of flame retardancy via the incorporation of nanomaterials, some of them showed negative effects of using them [134,135,136]. Recently, researchers turned to the simultaneous use of nanomaterials and FRs to improve the flammability of polymer nanocomposites. Enormous studies have been conducted about the combination of nanomaterials and FR compounds, which showed synergistic effects in different properties of nanocomposites [137,138,139]. Exploiting the combination of these nanoscale materials not only reduces the loading of nanomaterials and FR additives but also improves the mechanical and flame retardancy properties simultaneously.

### 4.1. The Role of Nanomaterials in Improving Flame Retardancy of PE Systems

Szustakiewicz et al. investigated on flame retardancy of HDPE/clay nanocomposites with MPP and APP FRs. They used two different types of organoclay: hydrophobic and hydrophilic. Based on the results of two flammability test methods (LOI and CCT), the simultaneous addition of organoclay and FRs to HDPE simultaneously results in a synergistic effect of flame retardancy. In this case, the LOI increases because of two factors, firstly, hydrophobic clay forms a reinforced structure that hinders the heat transfer of heat and secondly, APP intumescent char formation. As a result, the combination of these two effects makes the material burning more slowly [140].

**Figure 6:**
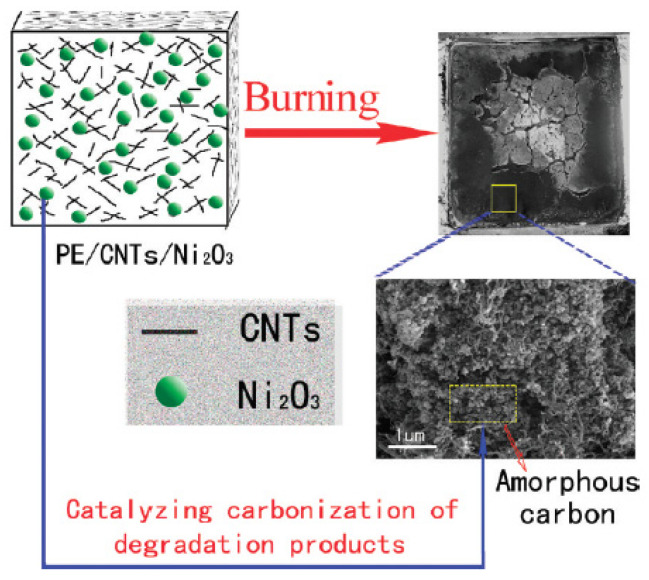
Schematic representation of the synergistic effect mechanism between Ni_2_O_3_ and MWCNTs on enhancing the flame retardancy of LLDPE, reprinted with permission from Ref. [142].

Chuang et al. also found that the incorporation of nano-dispersed layered silicate and low smoke non-halogen (LSNH) FRs to the EVA/HDPE polymer blend caused a synergistic effect on the flame retardancy and smoke suppressing. According to the results, during combustion, the HRR of the FR-EVA/LDPE-n nanocomposite is 40% lower than the FR-EVA/LDPE polymer blend. Furthermore, they investigated the effect of organoclay contents on the flammability of FR-EVA/LDPE-n. It was found that there is an optimum loading of organoclay (3 phr), where the nanocomposite has the highest performance in flame retardancy [141]. In another study by Yu et al., the effects of adding MWCNTs and Ni_2_O_3_ on the flame retardancy performance of LLDPE were investigated. The results of CCT show a synergistic effect of a combination of MWCNTs and Ni_2_O_3_ in improving the flame retardancy of LLDPE, such that nanocomposite containing 3 wt.% MWCNTs and 5 wt.% Ni_2_O_3_ shows 73% reduction in PHR rate compared to LLDPE and the yield of residual char is 13.7%. The improvement of flame retardancy of LLDPE by incorporating MWCNTs and Ni_2_O_3_ was attributed to the physical effect of MWCNTs (formation of a network like structure because of the good dispersion of MWCNTs), chemical effect of Ni_2_O_3_ (catalytic carbonization), and the combination of physical and chemical effect. Figure 6 schematically illustrates the aforementioned mechanisms [142].

**Figure 7:**
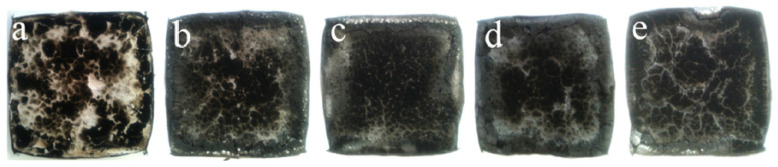
Photos of the residues after cone testing (100 ×100 mm^2^) of PE/ATH/GNPx, (**a**), x = 0.2 (**b**), x = 0.5 (**c**), x = 1.0 (**d**), and x = 1.5 (**e**), reprinted with permission from Ref. [143].

Han et al. applied different contents of well-exfoliated graphene nano-platelets to enhance flame retardancy of polyethylene/alumina trihydrate (PE/ATH) composites and showed the addition of 0.2 wt.% of GNP decreased the PHR by 18% of that of the PE/ATH composite. A possible explanation of this behavior is a char layer of GNPs acting as a heat shield and a barrier against mass transport. Images of the PE/ATH/GNPx (x, contents of GNP) after cone testing are represented in Figure 7.

Table 5 represents some results about the influence of nanomaterials on the flammability of PE nanocomposites.

### 4.2. Incorporation Methods of Nanomaterials in Polymer Matrices

A variety of methods are applied for the incorporation of nanomaterials into PE, depending on the nature of the nanomaterials [149]. These methods include in situ polymerization, solvent casting, and melt mixing, which will be elaborated in the following sections.

#### 4.2.1. In Situ Polymerization

In this method, at first nanomaterials and monomers are mixed in a solvent with a proper shear rate, which obtains a stable suspension [150]. In this stage, some interfacial agents are added to enhance the stability of the mixture. Then, the obtained mixture is fed to the reactor where the processing conditions mostly are the same applied to the synthesis of the base polymer. When the polymerization is complete, the solvent is removed [151]. This method is used for a wide range of polymer nanocomposites.

#### 4.2.2. Solvent Casting

This method is mostly used in cases that there is not enough dispersion of nanomaterials in the polymer [152]. In other words, the thermodynamic affinity between polymer and filler is not favorable for homogeneous dispersion. Therefore, the most important step of this method is the breaking of the agglomerates of nanomaterials, and ultra-sonication is the best method to achieve this goal. This process is especially suitable for the exfoliation of clay layers and thermosetting polymers [153,154]. To conserve dispersed/intercalated/exfoliated structure, some interfacial compatibilizers, such as maleic anhydride grafted polymers, are used.

#### 4.2.3. Melt Mixing

Melt compounding is the most common method for the production of nanocomposites because of the simplicity and availability of equipment [155,156]. Depending on the amount of the product, internal mixer and twin-screw extruder are used for processing. There are two major methods of feeding for melt compounding: direct and master-batch which the later method is more common. In the master-batch feeding method, first, a concentrated master-batch of filler is prepared and then is diluted in the base polymer [157]. To obtain a good dispersion of filler in polymer and improve compatibility among components, some grafted polymers are utilized [158]. The amount of shear rate, mixing time, temperature, and design of the screw profile can determine the final microstructure and properties of the nanocomposites [159,160].

## 5. Summary and Perspective

Flame retardancy of polymeric materials has become an area of keen interest recently following the rampant fire outbreaks. This research focused on the FRs and different application mechanisms available for the treatment of PE and its grades. In this study, a list of phosphorus, melamine, nitrogen, inorganic hydroxide, boron and silicon-based retardants with their loading amounts and effects on the grades of PE have been presented. The desirable FRs are the additives that presents a balance between fire resistivity and maintaining or improving the mechanical properties of the composite. It was realized from the research that the addition of FRs such as POSS, nanoclays, Organopalygorskite (OPGS), Molybdenum sulfide (MoS_2_), MH, TiO_2_, etc., greatly improved the fire and mechanical properties of the PE samples. Possible concerns for future research should be to investigate the effect of FR polymers on the environment. Most of the studies analyzed failed to assess the effects of the additives on the environment. Depending on the lifetime of FR polymers, for the short lifetimes, the biodegradability of polymer is an important issue, and the recyclability of FRs is an important factor for the polymers with a long lifetime. The innovative natural sources for the polymer FR additives are highly recommended possibilities for decreasing environmental issues. Moreover, during burning, some additives of the FRs can produce toxic compounds that must be controlled or even substituted. Addressing these concerns will be a step towards the betterment of the circular economy model.

## Figures and Tables

**Table 1:** Different properties of PE’s grades [19,20,21].

PE Grades	Structure’s Description	Density (g/cm^−3^)	Crystallinity	LOI (%)	Thermal Conductivity (W/m·K)	Melt Temperature (°C)	Mw (g/mol)
LDPE (Low-density PE)	Branched structure containing long and short chains	0.915–0.932	Lower degree of crystallinity	17–18	0.32–0.35	105–115	<50,000
LLDPE (Linear low-density PE)	Branched structure containing short chains	0.910–0.930	Slightly higher than LDPE	17–18	0.35–0.45	120–130	<50,000
HDPE (High-density PE)	Linear structure	0.940–0.970	Higher degree of crystallinity	17–18	0.45–0.5	128–136	Up to 200,000

**Table 2:** Recent studies on flame retardancy of PE using phosphorus, melamine, and nitrogen.

Polymer Matrix	Additive(s)	Mechanism(s)	Result(s)	Ref.
HDPE	CUMP	Char formation, Emission of non-flammable gases	-Reduction of decomposition rate -Increasing the yield of residue -Reduction of heat release rate (HRR) -Increasing the time of burning	[4]
HDPE	APP THEIC	Char forming	-HDPE/APP/THEIC showed the heist LOI value -IFR incorporation significantly declined the PHR rate of HDPE	[24]
LDPE	Carbonization agent, APP, MP	Char formation, Physical barrier creation able to deactivate the oxidation-active centers of carbon	-Optimal flame retardancy belongs to carbonization agent/APP/MP with the weight ratio of 7:7:1 -Increasing the maximum temperature of the decomposition peak of LDPE	[10]
LDPE	APP, Pentaerythritol (PER), Salt of MP, Dibromoneop-enty Glycol (DBNPG)	Char forming, Thermal barrier	-Improving the char layer -Thermal barrier behavior enhancement -Increase in melt viscosity with proper amount of DBNPG	[25]
PE	Poly (piperazine methylphosphonic acid pentaerythritol ester)	Char formation, Exert condensed phase	-Improvement the residual mass and thermal stability -Reduction in HRR, THR -UL-94 V0 rating	[26]
LDPE	APP, PER, MLM	Char formation	-Increasing in thermal stability	[27]
LDPE	PSPHD-SEP	Vapor phase radical-trapping effect	-Reduction in PHR rate, THR -Improving the thermal stability -Increasing the LOI value (21%) compared to neat LDPE -UL-94 V-2 rating	[28]
HDPE	10 wt.% of mono ammonium phosphate (MAP), ammonium zeolite (AZ), and microcrystalline cellulose (MCC)	Char forming	-Slowing down the burning rates of HDPE/MAP10 and HDPE/MCC/MAP5/AZ5 composite by 64% and 62%, respectively -Improving the LOI level and char forming by incorporating FRs	[29]
LDPE	THEIC, microencapsulated ammonium polyphosphate (MCAPP)	Formation of a compact char	In the composite with MCAPP/THEIC (2:1): -Achieving V-0 rating of UL-94 -Reduction in PHR rate and THR by 74.8% and 71.9%, respectively compared to pure LDPE -Enhancing thermal stability at high temperature	[30]
PE/Wood Flour (WF)	APP	Performance of WF as the charring agent with incorporation IFRs	-Achieving V-0 rating of UL-94 -Positive effect of IFRs and WF to control the fire spreading and the risk of combustion -Reduction in PHR rate of the composites containing IFRs and compatibilizers	[31]
PE	Phenyl phosphinic arid di-4-[1-(4-pheny phodphonic acid monophenyl ester-yl)-methyl-ethyl] phenyester dimelaminium (PDEPDM)	Char forming	In composite containing 32 wt.% PDEPDM: -Achieve in V-0 rating of UL-94 -Improvement in LOI, formation of char residue -Increase in char yield from 0.08 wt.% for neat PE to 5.17 wt.% for composite containing 40 wt.% PDEPDM at 800 °C -Reduction in tensile and impact strength	[32]
LDPE	Expandable graphite (EG), Ethylenediamine phosphate (EDAP), 3,5-diaminobenzoic acid phosphate (DABAP)	Releasing CO_2_ gas acting as an effective charring effect	-Substantial reductions in PHR rate for all flame-retarded samples -Decreasing the mass loss rate by adding intumescent additives	[33]
LLDPE	MLM salt of pentaerythritol phosphate montmorillonite (MPPM)	thermally stable char forming	-Enhancing the char formation and the thermal stability of LLDPE at high temperatures -Substantial reduction in PHR rate, THR, mean mass loss rate, and fire growth rate index -Achieving V-0 rating in UL-94V test	[34]
LLDPE	MLM salt of chitosan phosphate (MCHP), Organically modified montmorillonite (OMMT)	-Char forming	-Increasing the char residue -Improving the thermal stability -Reduction of PHR rate, total heat release (THR), CO, CO_2_ emissions and fire growth index (FGI)	[35]
HDPE	APP, PER, modified porous mesostructured silica (SBA-15)	Intumescent char layer formation	-Better flammability characteristics at low SBA-15 loadings (<2 wt.%) -Enhancement in fire properties affected by formation of crystalline silicone phosphate barrier	[36]
LDPE	RP, APP	Intumescent char layer formation	-Increase in LOI value from 17.5 to 24.2 by addition of 30wt% APP -The highest LOI value of 27.2 and UL-94 rating of V0 at ratio of 5:1 (APP:RP) -Increase in the gas phase action by the addition of RP	[37]
HDPE	APP, MLM	Intumescent char layer formation	-Improvement in the composite’s tensile strength and combustion process by FR loading’s increase -Improving the thermal stability and char formation’s promotion by FRs	[38]
PE	Pentaerythritol phosphate nickel salt (PPNS), APP	Intumescent char layer formation	- LOI value increased from 30% to 34% -Reduction in total HRR by 46.3% and 51.9% -Reduction in average mass loss rate by 40.6 and 87.5%	[39]
HDPE/WF	APP, Aluminum trihydroxide (ATH), SiO_2,_ CaCO_3_	Char forming	-Increase in both mechanical and fire properties by using nanofiller additive -Combination of APP and SiO_2_ showed the highest LOI value, and the lowest HRR	[40]
PE	DABAP, EDAP, EG	Char forming	-Higher decomposition temperature was attributed to DABAP -The best char yields was belonged to PE/DABAP -PE/EDAP/EG showed the best flame retardancy behavior	[41]
HDPE/WF	APP	Char forming	-APP decreased HRR and total smoke values of system -The heat of ignition remained constant -Maximum reduction of HRR obtained by increasing the amount of APP to 4 wt.%	[42]
HDPE	Phosphorous–nitrogen-based charring agent (PDTBP), APP	Intumescent char layer formation	-UL-94 V-0 rating -Low migration percentage (2.2%) -Decrease in PHR rate, THR, and fire hazard value -High tensile and flexural strength	[43]
LLDPE	MLM salt of montmorillonite phosphate (MMP), zinc borate (ZB)	Char layer formation	-Increasing in thermal stability and char formation -Reduction in PHR rate, mean HRR, THR, and mean mass loss rate -Reduction in the fire risk -UL-94 V-0 rating for the composite with 30 wt.% MMP and 5 wt.% ZB -Highest char residue formation for the composite with 32 wt.% MMP and 3 wt.% ZB -Max. fire performance index (142%) for the system with only MMP (40 wt.%)	[44]
Ethylene-vinyl acetate (EVA)/LLDPE	MLM, TRZ, and Bentonite Clay	Strengthening the protective char barrier produced by ATH	E-PE/120ATH in comparison with the conventional E-PE/185ATH achieved: -Self-extinguishing behavior (UL-94 V-0 rating) -Reduction in the stiffness and improvement in elongation at break, Composites with TRZ and clay showed 23% reduction in PHR rate and 11% in smoke production	[45]
LDPE/WF	APP, WF	Char forming	Increasing the LOI value from 17.5 to 24.2 with addition of 30 wt.% m-APP 25% reduction in THE in the LDPE/WF/APP.	[46]

**Table 3:** Recent studies on flame retardancy of PE using inorganic hydroxides.

Polymer Matrix	Additive(s)	Mechanism(s)	Result(s)	Ref.
HDPE	MH, Modified MH	Char forming with both MH and modified MH	-The flame sustainability of HDPE/modified MH was higher than HDPE/MH -The flame retardancy behavior of HDPE/modified MH did not enhance compared to HDPE/MH	[67]
HDPE	ATH, MH	Endothermic decomposition reaction and heat absorption	-The HDPE/ATH/MH system demonstrated the lowest value of PHR rate -The lowest amount of THR belonged to HDPE/ATH/MH system -Combination of ATH and MH indicated the significant non-flammability behavior	[68]
LDPE	Zn/Al, Ni/Al, Co/Al	Char forming	-The incorporation of Zn/Al-LDH and Ni/Al-LDH with LDPE showed more decrease of flammability compared to Co/Al-LDH incorporation -The composite containing LDH alternated with organic onions indicated more flammability reduction in comparison with composite containing inorganic anions	[69]
LDPE	ATH, MH, Ferric oxyhydroxide (FH)	Char forming, Restriction of oxygen diffusion	-The higher LOI value of composites containing ATH compared to composites containing MH and FH -LDPE/MPP/Starch (ST)/ATH system indicated the more protective charred layer with smaller pores on it compared to other systems	[14]
LDPE/EVA	Organopalygorskite (OPGS), Molybdenum sulfide (MoS_2_), MH	Char formation	-Increasing the LOI value (26%) -Reduction in the burning rate (66%) and PHR rate (83%) compared neat polymer blend -Indicative the UL-94 V-0 rating	[70]
Paraffin/HDPE	MH, ATH, EG	Char formation, physical barrier	-Increasing in thermal stability and carbonization ability -Increasing the amount of char residue -Reduction in the THR and PHR rate	[71]
PE/PCS	MH	Exert condensed phase, barrier effects of char formation	-Improving thermal stability -Forming the multi-layered char structure	[72]
EVA/LDPE	ATH, Magnesium hydroxide sulfate hydrate (MHSH)	Char forming	-Improving thermal stability -Reduction in thermal degradation rate in the temperature ranges of 410 °C∼510 °C -Indicative V-0 in UL-94 test	[73]
LDPE/EVA	MH, Keratin fibers (KF), deoxyribose nucleic acid (DNA)	Char forming	-Increasing the LOI up to 24.5% -Reducing the HRR by 82% compared to PE/EVA sample with 55 wt.% MH	[74]
LDPE/EVA	MH, TiO_2_	Char forming	-Reaching to V-0 with LOI value of 24.9% -Increasing mass residue from 5 wt.% for blend to 25 wt.% for the composite containing both FRs -Increasing tensile strength and modulus of LDPE/EVA blend from 6.4 MPa to 7.1 MPa and 127 MPa to 133 MPa respectively by incorporation of both FRs -Improving impact strength from 27.8 to 35.2 KJ·mm^−2^	[75]
LLDPE	MH, SiO_2_	Char forming	-Improving thermal degradation resistance and the LOI value -Reduction in PHR rate and THR	[76]
LLDPE	Huntite and hydromagnesite (HH)	Char forming	-Increase in value of LOI and elastic modulus -Reduction in the horizontal burning rate, tensile strength, and elongation at break	[77]
HDPE/LDPE/Nylon 6	MH, MWCNT, Kenaf fiber	Char forming	-Increasing the tensile strength value by 50% at 0.5/0.5 wt.% loading of Mg(OH)_2_/MWCNT compared to composite without filler -Reduction in PHR rate with addition of Mg(OH)_2_/MWCNT	[78]
HDPE/WF	MH, 1,2-bis(pentabromophenyl) ethane, Aluminum hydroxide	Char forming	-Significant decrease in the HRR and THR -Best fire resistance for composite containing 1,2-bis(pentabromophenyl) ethane	[79]
MDPE	MH, Calcium-based hydrated minerals	Formation of cohesive CaCO_3_ combustion residue	-Reduction in PHR rate for Ca-based composites -Generation of an intumescent mineral residue during the combustion by calcium hydroxide	[80]
LDPE	MH, Montmorillonite (MMT)	Char forming	-Higher interlayer spacing is observed for organosilylated clay (SC1) compared to original MMT -Improving thermal stability compared to commercial organoclays	[81]
LDPE	Azocyclohexane (AZO), Bis(cyclohexylazocyclohexylmethane) (BISAZO), FlameStab^®^ NOR116, Magnesium dihydroxide (MDH), Luvogard MB81/PE	Intumescent char layer formation	-Better performance in flame retardancy when using AZO and BISAZO compared to the other additives	[82]
LDPE/EVA	Hexaphenoxylcyclotriphosphazene, Mg(OH)_2_, Al(OH)_3_	Char forming	-Blends showed better flame retardancy when composited with Mg(OH)_2_ and Al(OH)_3_ -The maximum specific optical density is reduced from 370.65 to 91.72 -An increase in the residual volume and compactness of solid residue surface layer based on SEM morphology is observed	[83]
LDPE	ATH, EVA	Char forming	-Flame resistance of EVA/LDPE/ATH blends is slightly enhanced after γ-irradiation -Increase in the cross-linking density caused an enhancement in electrical and thermal properties -γ-irradiation delayed the thermal degradation process of EVA/LDPE/ATH blends	[84]
LDPE/Cross-linked polyethylene (XLPE)	MMT, MH, LDPE-g-MA	Char forming	-The increase in the tensile and impacts strengths induced by the addition of clay and LDPE-g-MA -Thermal stability at high temperatures is enhanced due to the increase in char residual of nanocomposites -XLPE nanocomposites showed efficient level of flame retardancy	[85]
MDPE/EVA	MDH, Hydrated lime, Hydrated dolomitic limes	Intumescent char layer formation	-Ca-based MDPE composites depicted similar rates of PHR with MDH composite -Lower PHR rate observed for Ca-based fillers in EVA compositions -The formation of an intumescent cohesive residue in the combustion process is induced by an effective role of calcium di-hydroxide	[86]
HDPE	ATH, ZB	Char forming	-2 phr organo-clay additive is used to achieve V0 rating -FR materials with high processability and mechanical properties is obtained when using HDPE rendering	[87]
LLDPE/Ethylene-acrylic acid (EAA)	MH	Char forming	-Addition of EAA improved LOI value of LLDPE/EAA/MH from 28% to 30% -Reduction of HRR and SPR values was occurred because of the acceptable dispersion of MH -Improvement of thermal oxidative stability of LLDPE/EAA/MH due to the EAA presence	[88]
LLDPE	CaCO_3_, MgCO_3_, Talc	Intumescent char layer formation	-HRR peaks were considerably reduced with incorporation of all mineral fillers -Improvement of nanoparticle dispersion in LLDPE by stearic acid	[89]

**Table 4:** Recent studies on flame retardancy of PE using boron and silicon based FRs.

Polymer Matrix	Additive(s)	Mechanism(s)	Result(s)	Ref.
PE	ZB, Phosphorus–Nitrogen (DOPO-N)	Exert condensed phase and gas phase	For the PE/20%ZB/10%DOPO-N composite: -Increasing in thermal stability -Reduction in PHR, THR, average heat combustion, and FGI	[104]
HDPE	Fullerene (C60), Decabromodiphenyl oxide/Sb_2_O_3_ (brominated FRs)	trapping radical ability in condensed phase and gaseous phase by C60 and BFR, respectively	-Improving the thermal and thermo-oxidative stability of HDPE/BFR blends by adding C60 -A remarkable reduction in PHR rate especially at higher concentration of C60	[105]
HDPE/WF	1,2-bis(pentabromophenyl) and ethylene bis(tetrabromophthalimide), and nanoclay, MAPE as compatibilizer	Trapping the free radical produced from WF by Bromine radicals Char forming by WF and nanoclay	-Decreasing the composite strength by adding FRs -Synergistic effect in 1,2-bis(pentabromophenyl)-clay-MAPE system by reducing PHR rate and increasing thermal stability	[106]
HDPE	WF, BA, borax (BX)	Char forming	-CCT showed that the addition of BA/BX improved the fire performance of the samples -Increasing the ratio of BA/BX has a negative effect on ignition time, HRR, smoke production rate, and specific extinction area	[107]
HDPE/EVA	Two different particle sizes of EG	Char forming	-According to TGA and CCT tests, thermal stability and fire resistance of HDPE/EVA blend considerably increased due to the existence of EG -EG incorporation decreased the mechanical properties	[108]
mLLDPE/(NR/ENR-50)	ZB	Char formation	-Improvement in crystallinity of all the blends due to ZB presence and the best crystallinity was obtained at 6 phr ZB blend -Increasing the thermal stability of NR because of ZB incorporation -The best thermal stability was achieved at 8 phr ZB blend -Incorporation of ZB enhanced the LOI value of mLLDPE	[109]
HDPE	Modified Clay	Decomposition of fillers and char layer formation	-The decrease in PHR from 13 to 62% by adding 3, 5 and 7 wt.% of each PFS1 or PFS2 and their OMMTPFS1 and O-MMTPFS2 -62.41% reduction in PHR rate for the composite containing 7 wt.% of O-MMTPFS2 -TTI was higher or similar to initial HDPE for all samples -Decrease in the fire growth rate for all composites by increasing the filler loading	[110]
LLDPE	Aerosil^®^ r974 organically treated fumed silica (Ar974) in combination with Al hydroxide Alufy^®^ 2 (AF) or Mg hydroxide Hydrofy^®^ G1.5 (HF)	Char Formation	-Both PE/HF/Ar974 composites with 20 wt.% HF and (2 or 5 wt.% Ar974) self-extinguished (LOI values were 31.9% and 35.2%, respectively) -Effect of nanosilica on decreasing the PHR rate is significant in synergistic systems -Composite containing 20 wt.% HF and 5 wt.% Ar974 showed best fire performance based on LOI and CCTs	[111]
HDPE	Aminosilane modified silica in combination with MWCNT	Char layer formation that can be promoted by MWCNT	-Composite with 5% MWCNT and no nanosilica represented the max. value of LOI: 26.0 (36.8% higher than that of neat HDPE) and the min. value of the PHR rate (54% reduction) -Increase in MWCNT loading decreased PHR rate -Lowest smoke production for the composite with only nanosilica and highest with the ones with only MWCNT -Higher MWCNT loading, thicker and more homogeneous char layer -Slight synergism between fillers	[112]
LDPE	4A zeolite	Intumescent char layer formation	-Enhancement in the LDPE/IFR’s LOI value -Successful passing in the UL-94 V-0 rating test for all composites -Improvement in the strength and compactness of the char surface	[113]
HDPE	SiO_2_ or CaCO_3,_ APP, PER	Intumescent char layer formation	-Sample composition has significant role in WPCs’ properties -Best properties obtained when using SiO_2_ as the filler	[114]
LDPE/EVA	Nanoclay, ATH, ZB	Char formation	-Using nanoclays improved many parameters of flammability including ignition time, FGI, and PHR -Nanoclays effects are intensified when combined with traditional aluminum hydroxide or aluminum hydroxide	[115]
LDPE	Fe-MMT, Fe-OMMT	Intumescent char layer formation	-Lower HRR and lower THR observed for LDPE/IFR/Fe-MMT compared to LDPE/IFR/Fe-OMMT for the same loading percentage	[116]
HDPE	APP, SiO_2_	Char formation	-Lower initial temperature and peak temperature of thermal degradation is achieved for RPC compared to wood-HDPE composites (WPC) -Introducing APP to RPCN expedites the thermal degradation of RPC -Better flame retardancy is observed for RPC	[117]
LDPE/EVA	OMMT, Piperazine spirocyclic pentaerythritol bisphosphonate) (PPSPB)	Intumescent char layer formation	-Thermal stability increased while flammability considerably decreased -PHR rate, THR, and average mass loss rate reduced significantly -The PHR rate of LDPE/EVA/PPSPB/OMMT showed 50% reduction compared to the LDPE/EVA blend.	[118]
Wood fiber-HDPE	Nano-SiO_2_	Char formation	-Reduced the HRR, THR, and total smoke release of wood fiber-HDPE composites -Tensile and flexural strength improved	[119]
HDPE/Wheat straw	Mg(OH)_2_, Nanoclay	Char formation	-Increasing the nanoclay and Mg(OH)_2_ content reduced the burning rate, tensile and impact strength of the samples -Increasing the nanoclay weight percentage increased the tensile modulus and impact strength	[120]
PE	MMT, Sepiolite, POSS	Char formation	-HRR of CaSiEBA significantly increased after MMT nanofibers addition -Flammability retardancy of CaSiEBA and CaSiEMAA remained unchanged after sepiolite incorporation -Reduction of dripping was occurred due to the addition of only small amount of POSS -POSS enhanced HRR value of CaSiEMAA	[121]
PE	OMMT, Diphenylmethanamine spirocyclic pentaerythritol bisphosphonate (PSPD)	Intumescent char layer formation	-Combination of PSPD and montmorillonite (MT) improved the thermal stability of LDPE -The flammability of LDPE Extremely reduced due to the addition of PSPD/MT -51% decrease in the PHR rate of LDPE/PSPD/OMMT in comparison with LDPE	[122]
HDPE	MH, Aluminium hydroxide, EG, APP, PER, MMT	Char formation	-Improved flame retardancy behavior obtained by using APP/PER/MMT and APP/EG -Increasing the thermal stability of HDPE due to the FRs incorporation	[123]

**Table 5:** Effects of different types of nanomaterials on the flammability of PE.

Nanomaterial and Its Loading Amount	Types of FR and Its Loading	Result(s)	Ref.
Ce-MWCNTs, 3 wt.%	Brominated FR, 10 wt.%	25% reduction in PHR rate observed from CCT, improved the UL-94 from V-2 to V-0	[144]
Nano-SiO_2_, 6 wt.%	APP, 8 wt.%	42% and 44% reduction in average HRR and PHR rate, respectively, 78% increase in TTI	[145]
Organic-modified montmorillonite, 10 wt.%	MHSH, 30 wt.%	84% reduction in PHR rate and increase in t_ign_ observed from CCT.	[146]
Organic-modified montmorillonite, 5 wt.%	IFRs, 15 wt.%	51% reduction in PHR rate observed from CCT	[122]
Halloysite nanotubes, 2 wt.%	IFRs, 28 wt.%	92% and 75% decrease in PHR rate and THR, respectively.	[147]
Graphene, 1 wt.%	Brominated polystyrene/antimony trioxide, 6.2 wt.%	Increase LOI value from 23.4% to 24.1%, change UL-94 grades from NG to V-2.	[148]
